# The Chicago Conference on Medical Education

**Published:** 1907-06

**Authors:** 


**A Monthly Review of Medicine and Surgery.**

*EDITOR:*

WILLIAM WARREN POTTER, M. D.

All communications, whether of a literary or business nature, books for review and exchanges, should be addressed to the editor 238 DELAWARE AVE., BUFFALO, N. Y

VOL. LXII. JUNE, 1907. NO. 11

### The Chicago Conference on Medical Education.

THE third annual conference of the Council on Medical Education of the American Medical Association was held at Chicago, April 29, 1907. A large number of persons interested in medical education from all parts of the country was present. In addition to the six members of the council eighty-four delegates were in attendance representing twenty-two state medical examining boards, eight state medical societies, the three branches of the government service, three college associations, and seven colleges of liberal arts. The entire conference, which lasted until near 6 o'clock p.m., was one of unusual interest and reflects credit not only upon the council itself, but upon all who have contributed by pen or speech to a betterment of medical education on the lines laid down by the council.

One of the most important and far reaching measures adopted by the conference is the inspection of the medical colleges in the United States. This inspection was voted at last year's meeting and the chairman, Dr. Bevan, in his address gave a synopsis of the inspection report, which disclosed an astonishing condition of affairs among the medical schools of the country. It is surprising after all the warnings given that so many colleges should lag behind in the march toward higher ideals.

Dr. Bevan's report disclosed the fact that nearly one-half of the medical schools in the United States fell below 70%, that being the minimum marking of acceptability. In other words, of the 160 schools in the United States 81 were above 70%; 47 were between 50% and 70%, while 32 were below 50%. Schools graded above 70% are considered as worthy of recognition.

The basis of the inspection marking was that adopted by the civil service and the grading was obtained under the following general heads:

1. Failures of graduates before State Examining Boards.
2. Evidence of a satisfactory preliminary requirement.
3. Character and extent of the college curriculum.
4. Number, condition, and utility of college buildings.
5. Laboratory facilities and instruction.
6. Dispensary facilities and instruction.
7. Hospital facilities and instruction.
8. Extent to which laboratories are officered by men devoting their entire time to teaching and research.
9. Extent to which the school is conducted for profit directly or indirectly rather than for teaching.
10. Medical library, museum, stereopticons, charts, etc.

The fairness of the inspection cannot be questioned. The markings in each instance are liberal and the classifying of the colleges has been a matter of great deliberation. Chairman Bevan, in his address, pointed out the following facts as shown by the inspection:

1. Of the 160 schools, 81 received markings above 70, 47 markings between 50 and 70, 32 below 50.

2. It was clearly shown by this inspection that medical schools conducted solely for profit are a menace and should not be recognised.

3. Night schools attempting to educate a student in the hours from 7 to 10 p.m., usually after the student has devoted his day to some occupation in which he earns his living, should not be recognised by any state board, especially when it is found in the good schools that four years of nine months six days in the week from 8 a.m. to 5 p.m., is barely sufficient time to devote to a medical course.

4. Many schools are conducted for the purpose of preparing a student to pass a state board examination and not with the object of making him a competent practitioner.

5. A student can be prepared in a quiz class in a comparatively short course to pass a written examination before a state board and yet be absolutely ignorant of laboratory work, dispensary or hospital work, and utterly incompetent to begin the practice of medicine. This fact shows the necessity of inspecting the schools as to the actual character of their work, of making the written examination very practical and of including a practical examination in the laboratory and possibly later on patients in the state board examination for licensure.

6. The most important fact brought out in this inspection is this: In this country we need money for medical education. It costs more to educate a medical student than he can pay in the way of fees. Medical education must secure state aid and private endowment.

7. Every state university now in existence or established later should have a strong, liberally supported medical department. No better investment can be made by any state than the establishment and support of such a medical department. Such a department, at least the clinical years, should be in the largest center of population in the state, and in close touch with the various departments of the state board of health.

8. The public must be taught the necessities and the possibilities of modern medicine, and philanthropists shown that medicine well deserves the same support that has been given to theology, to colleges of liberal arts, to libraries, etc. Philanthropists will not endow medical schools which are private corporations and conducted for profit. Such schools should change their organisations so that they can ask for and secure necessary endowments.

9. It is in the power of the state boards and the organised profession to place American medicine on an acceptable plane.

10. Already about 50 schools have agreed to require, by 1910 or before, one year of university physics, chemistry and biology, and one language as a preliminary education before matriculating in medicine.

11. It is necessary to agree on a standard of what shall constitute a medical college in good standing. If, as a result of this conference, the delegates from the state boards agree to require within a reasonable time the following standard of our medical schools, we should soon place ourselves on a plane equal to that of any country in the world:

(a) Preliminary education sufficient to enter the freshman class of our universities plus, after a date to be agreed on, one year of physics, chemistry and biology and one modern language. This preliminary education to be passed on by an officer selected by the state licensing board.

(b) Two years of study, largely laboratory work in anatomy, physiology, pharmacology and pathology in well equipped laboratories, officered by trained men devoting their time to those subjects.

(c) Two years of clinical work largely in dispensaries and hospitals, the dispensary material in the proportion of at least 10,000 per year for 100 students in the senior class and the hospital in proportion to a daily average of 200 patients to 100 students in the senior class.

(d) The curriculum and character of both laboratory and clinical work to be satisfactory to the state licensing board, which shall inspect the same each year.

The state medical examining boards were recognised by the conference, not only through the reports of the chairman and secretary, but in the debates that took place on these reports and on the other papers read, as the only legally constituted authority to deal with the questions involved, the function of all other bodies including the council itself, being merely advisory in character. It, therefore, behooves the state boards to take up these questions in all seriousness and to cooperate with the council in this most important, even vital matter. In order that efficiency may be maintained and success reached with reasonable promptitude it is essential that the present personnel of the conference shall continue intact, as far as possible. The representation from states and institutions not now in affiliation with it is of the greatest importance, and this should be urged by every consideration looking toward safeguarding the interests involved.

